# “I feel that life is meaningless”: Vietnamese adolescents' experiences of and reflections about interpersonal violence

**DOI:** 10.1017/gmh.2017.34

**Published:** 2018-04-03

**Authors:** M. T. H. Le, S. Holton, M. Kirkman, J. Fisher

**Affiliations:** Jean Hailes Research Unit, School of Public Health and Preventive Medicine, Monash University, Melbourne, Victoria, Australia

**Keywords:** Adolescents, interpersonal violence, low- and middle-income countries, mental health, poly-victimisation, Vietnam

## Abstract

**Background:**

The experiences of and reflections on interpersonal violence and victimisation among adolescents living in low- and middle-income countries are poorly understood. The aim was to describe Vietnamese adolescents’ reflections on their experiences of victimisation.

**Method:**

A self-completed, cross-sectional survey investigating exposure to violence among high school students in Hanoi, Vietnam was conducted during 2013–2014. The last section invited participants to write about any of the matters covered in the questionnaire. Thematic analysis was conducted on these free-text comments.

**Results:**

A total of 73/76 eligible students participated in the pilot and 1616/1745 in the main survey. Of these, a total of 239 records with free-text comments were analysed. Students described experiences of violence occurring at home, at school and in the community. Experiences of violence led to sadness, loneliness, having extremely negative thoughts about the value of life, and suicidal ideas. Adolescents’ experiences occurred in the context of poor parent–adolescent and teacher–student relationships, particularly concerning dissatisfaction with academic performance. Adolescents wanted to be trusted, to be given more autonomy, and to improve their relationships with parents and teachers.

**Conclusions:**

Vietnamese adolescents experience various forms of victimisation, which are detrimental to their health and wellbeing. Understanding of their experiences of and perceptions of violence and its impact on their health and wellbeing is important in the prevention of violence against young people in Vietnam.

## Introduction

Interpersonal violence is among the top five causes of death and leading contributors to the burden of disease among adolescents (World Health Organisation, 2014). Intentional and unintentional injuries were estimated to account for 28% of deaths among 10–14-year olds and 44% among 15–19-year olds in 2012 (United Nations Children's Fund, 2014).

Most of the world's adolescents live in low- and middle-income countries (Gupta *et al.* 2014), and experiences of violence are prevalent among them. Of 124916 children and adolescents living in 28 low- and middle-income countries who participated in the UNICEF Multiple Indicator Cluster Surveys, Akmatov (2011) reported that more than 92% had experienced psychological abuse, 81% moderate physical abuse (shaking, hitting or slapping limbs) and 61% severe physical abuse (slapping face or head; being hit with a hard object).

Qualitative research enables a deeper and more detailed understanding of participants’ experiences in comparison to quantitative research (Hammarberg *et al.* 2016). There is evidence from qualitative research in high-income countries that adolescents may hold different opinions about what constitutes violence and its impact on them (Kulig *et al.* 2006; Marsh *et al.* 2007).

Focus group discussions among New Zealand students revealed that one form of violence, aggression, was described by the students as being on a continuum that included not only physical fighting, but also play fighting, scratching, slapping or hair pulling and verbal aggression among girls (Marsh *et al.* 2007). Canadian rural youths aged 11–19 emphasised in interviews that psychological violence was more harmful than physical violence because emotional scars are difficult to heal (Kulig *et al.* 2006). Qualitative findings such as these may inform subsequent quantitative research that is meaningful to young people and relevant to the local context.

Vietnam is a lower-middle-income country with more than 30 million children and adolescents (General Statistics Office of Vietnam, 2011). Experiences of victimisation and maltreatment are widespread among them (Nguyen *et al.* 2010; Le *et al.* 2015). Those who are victimised are more likely to be girls, to live in a rural area, to live with a step-parent, and to have parents with a low level of educational attainment (Le *et al.* 2015). These experiences have also been shown to be associated with lower self-esteem (Nguyen *et al.* 2010), poorer emotional and cognitive functioning (Tran *et al.* 2017), and increased symptoms of common mental disorders and suicidal behaviours (Le *et al.* 2012, 2016*b*, 2017). However, there are few data about adolescents’ experiences of and reflections on violence. In focus group discussions about child maltreatment with teachers and 12–17-year-old students in rural and urban areas of Vietnam, Nguyen (2006) identified eight illustrative themes including an understanding of what constituted child maltreatment and its prevalence, use of corporal punishment, perpetrators, and source of information about and methods to prevent child maltreatment. These themes informed a subsequent quantitative survey about child maltreatment among young people in Vietnam, conducted by Nguyen *et al*. (2010). Nevertheless, the experiences of and reflections on the violence of this vulnerable population group in Vietnam are not well understood.

Current legislation in Vietnam, in particular, the *Law on the care and protection of children*, recognises the importance of preventing violence against young people. However, there is no national program to prevent violence or to support young victims of violence. The lack of in-depth understanding of adolescents’ perspectives on violence limits the development of effective prevention and support programs in Vietnam.

The aim of this study was to describe Vietnamese adolescents’ reflections on their experiences of victimisation.

## Methods

Seeking free-text comments at the end of a self-report survey is common research practice. Although not all participants contribute free text, these data provide valuable insights into experiences, reveal new perspectives, and are suitable for content, thematic and narrative analyses (Garcia *et al.* 2004; Rich *et al.* 2013; Dixon *et al.* 2014). A cross-sectional, self-completed, anonymous survey about experiences of poly-victimisation, which included an invitation to provide free-text elaboration, was completed by high school students in Vietnam and has been described elsewhere (Le *et al.* 2015).

*Study setting*: The survey was conducted in urban and rural districts of Hanoi, Vietnam, from October 2013 to January 2014.

*Participants*: The students were in grades 10–12 at ten high schools of different types (public, private, and centres for continuing education).

*Measures*: The primary objective of the study was to describe experiences of poly-victimisation, using the translated and culturally verified for Vietnam Juvenile Victimisation Questionnaire Revision Two (JVQ-R2) (Finkelhor *et al.* 2005), and the mental health and quality of life of adolescents in Vietnam.

Information about socio-demographic characteristics, academic pressure and performance, relationship with parents, involvement in health risk behaviours, symptoms of common mental disorders, perceived general health and health-related quality of life was sought using standardised and study-specific fixed-response questions (Le *et al.* 2015, 2016*a*, *b*).

The final section of the questionnaire invited free-text comments with the question, ‘We do not have any more questions for you, but there may be other things you would like to tell us. If so, please feel free to write on the lines below’.

### Procedure

The questionnaire was translated, culturally verified in pre-testing, then pilo*t* tested for comprehensibility, acceptability and salience in a group of adolescents not attending the participating schools. After final elaborations to eliminate definitional ambiguity, the questionnaire was administered during a class period to students in the selected grades in ten high schools.

### Data analysis

The JVQ-R2 was scored using standard criteria. A total poly-victimisation score was calculated as the sum of all 37 items (‘yes’ coded as 1 and ‘no’ as 0). Students were divided into three groups: non-victims whose scores were 0, victims whose scores were 1–10, and poly-victims whose scores were 11 or more. Prevalence of different forms of victimisation and of poly-victimisation was calculated separately for those who provided free-text comments and those who did not. Chi-square and *t* tests were performed to compare the demographic and victimisation characteristics between these two groups. These analyses were conducted in IBM SPSS 20.0 (IBM Corp., 2011).

Thematic analysis (Braun & Clarke, 2006) was conducted on the free-text comments. ML read all comments repeatedly and recorded the themes in a Word document. All comments related to interpersonal violence were translated from Vietnamese into English by ML and the translation reviewed by two public health researchers bilingual in Vietnamese and English. Where there was no directly equivalent word between Vietnamese and English, the authors agreed upon the most suitable word after discussion. The original Vietnamese words are presented in brackets and the closest English words chosen are included in the translated quotes. Themes reflecting the participants’ experiences of victimisation, descriptions of their feelings and emotions, and reflection on their experiences, where available, were identified and the comments coded accordingly. Identified themes were organised into a hierarchy of themes and all free text reassessed to ensure that the themes were appropriate and comprehensive. The first author performed the initial analysis; results were refined after discussion among all authors.

### Ethics

All heads of participating schools and centres provided letters of approval for the study. Parental opt-out consent was sought for all students less than 18 years of age. Student participation was voluntary. Students’ consent to participate was implied in their completion of the questionnaire. Ethics approval for the conduct of the study was granted by the Institutional Review Board of the Hanoi School of Public Health (Application ID: 013-148/DD-YTCC) and the Human Research Ethics Committee of the Monash University (project ID: CF13/1762-2013000897).

## Results

A total of 1616 out of a potential 1745 students participated in the survey and 75 in the pilot study. Among these, 239 (15%) contributed free text. The students who contributed free text had a mean age of 16.5 years (minimum 14.9 and maximum 19.5); slightly more than half (53.1%) were female and nearly half (46.9%) lived in a rural area. There were no differences in age or residential areas between those who provided free text and those who did not.

Prevalence of different forms of victimisation and of poly-victimisation for the 239 students who provided free-text comments and the 1452 students who did not is presented in Table 1. Overall, compared to students without free-text comments, students with free-text comments were significantly more likely to have had lifetime experiences of any victimisation and poly-victimisation.

**Table 1.** Prevalence of different victimisation forms and poly-victimisation among those providing free-text comments and those who did not, in a sample of Vietnamese high school students

Victimisation form	Students with free-text comments (*N* = 239) % (95% CI)	Students without free-text comment (*N* = 1452) % (95% CI)
Conventional crime		
Robbery**	19.3 (14.3–24.4)	12.4 (10.6–14.1)
Personal theft***	53.8 (47.4–60.2)	41.3 (38.8–43.9)
Vandalism	51.3 (44.9–57.7)	46.7 (44.2–49.3)
Assault with weapon***	30.9 (25.0–36.9)	18.5 (16.5–20.5)
Assault without weapon*	56.5 (50.2–62.9)	48.9 (46.3–51.6)
Attempted assault	23.5 (18.0–29.0)	21.5 (19.3–23.6)
Threatened assault**	49.2 (42.8–55.6)	38.5 (36.0–41.1)
Kidnapping	2.1 (0.3–3.9)	1.5 (0.9–2.1)
Bias attack**	4.2 (1.6–6.8)	1.4 (0.8–2.0)
Child maltreatment		
Corporal punishment	67.1 (61.1–73.1)	61.3 (58.8–63.9)
Physical abuse by caregiver**	63.7 (57.6–69.9)	53.2 (50.6–55.8)
Emotional abuse**	44.1 (37.8–50.5)	33.8 (31.4–36.3)
Neglect***	20.2 (15.0–25.3)	10.7 (9.1–12.3)
Family abduction	5.9 (2.9–8.9)	3.5 (2.5–4.4)
Peer and sibling victimisation		
Gang or group assault*	24.8 (19.2–30.4)	16.1 (14.2–18.0)
Peer or sibling assault*	38.7 (32.4–44.9)	31.6 (29.2–34.1)
Nonsexual Genital Assault	12.6 (8.4–16.9)	10.7 (9.1–12.3)
Physical Intimidation by peers*	28.2 (22.4–33.9)	20.5 (18.4–22.6)
Relational aggression by peers ***	37.7 (31.5–43.8)	26.5 (24.2–28.8)
Dating violence	11.3 (7.3–15.3)	13.0 (11.3–14.8)
Sexual victimisation		
Sexual assault by known adult	8.0 (4.5–11.5)	5.6 (4.4–6.9)
Sexual assault by unknown adult	8.4 (4.9–12.0)	5.4 (4.2–6.5)
Sexual assault by peer/sibling	3.8 (1.3–6.2)	2.5 (1.7–3.3)
Forced sex	3.8 (1.3–6.2)	3.3 (2.4–4.3)
Flashing/sexual exposure*	19.7 (14.6–24.7)	13.2 (11.5–15.0)
Verbal sexual harassment***	18.8 (13.8–23.8)	10.2 (8.6–11.8)
Statutory rape& sexual misconduct*	10.5 (6.6–14.4)	6.2 (5.0–7.5)
Witnessing and indirect victimisation		
Witness to domestic violence**	17.7 (12.8–22.6)	11.3 (9.6–12.9)
Witness to parent assault of sibling	47.3 (40.9–53.7)	40.8 (38.2–43.4)
Witness to assault with weapon*	54.8 (48.5–61.2)	46.8 (44.2–49.4)
Witness to assault without weapon*	59.5 (53.2–65.8)	50.7 (48.1–53.4)
Burglary of family household*	50.2 (43.8–56.6)	41.3 (38.7–43.8)
Murder of family member or friend	10.1 (6.3–14.0)	7.1 (5.8–8.5)
Exposure to random shootings, terrorism or riots	7.5 (4.2–10.9)	5.6 (4.4–6.8)
Exposure to war or ethnic conflict	5.4 (2.5–8.3)	3.0 (2.2–3.9)
Family violence and abuse		
Parental Displaced aggression**	38.1 (31.9–44.3)	28.6 (26.3–31.0)
Other family violence exposure***	35.2 (29.0–41.3)	24.3 (22.0–26.5)
Internet harassment**	35.6 (29.5–41.7)	26.8 (24.5–29.1)
Poly-victimisation category***		
Non-victims	1.4 (0–3.0)	6.1 (4.8–7.4)
Victims (1–10 types)	51.2 (44.4–57.9)	64.1 (61.5–66.8)
Poly-victims	47.4 (40.7–54.2)	29.7 (27.2–32.3)

*p < 0.05 for comparison between students with free-text comments and those without.

**p < 0.01 for comparison between students with free-text comments and those without.

***p < 0.001 for comparison between students with free-text comments and those without.

Three main themes emerged from analyses of students’ comments: the forms of violence to which they were exposed, emotional and relationship consequences of experiencing violence, and the hopes and aspirations expressed by victims of violence.

### Theme 1: Adolescents’ exposure to various forms of violence

Students described diverse experiences of victimisation, including maltreatment and neglect at home, abuse at school, and sexual assaults by strangers. Some described poly-victimisation. It was notable that some forms of victimisation, including economic abuse, which were not in the JVQ-R2 were described.

Victimisation by parents included extreme control and prohibition of friendships.

Why for students in high school like us, having a relationship with a boyfriend is still considered to be rubbish (nhăng nhít, vớ vẩn) by parents? And it is discussed and prohibited in many ways? … Parents always think they're right, … and do not listen to what their children say. [15, female, urban]

Victimisation at school included fines or belittling and unfair treatment from teachers. Two male students reported having to pay fines whenever they made a mistake:

‘Each time [I] make a mistake at school or in the class, [I] have to pay fines’ [17, male, rural]

‘Each time the female teacher reminds me of the fine, I feel stressed to death (I do not have a cent).’ [18, male, rural]

Another female student described how she was treated unfairly and insulted by a math teacher:

I was HATED [by the math teacher]!!! In class, during examinations, I am always placed at the end of the class … and then got bad marks, insulting eyes and words from him. … I was named in front of the class, with uncomfortable sentences which could be considered insulting, by the maths teacher!!! [17, female, urban]

### Theme 2: Emotional and relationship consequences of experiencing violence

Witnessing and experiencing violence led to feeling scared, sad, and lonely. All students who witnessed verbal violence between parents said they were sad:

There were times when I saw my parents arguing with each other and I felt really sad. [16, male, rural]

Some also wrote that they were scared and concerned about what could happen, including a divorce between their parents. When domestic violence elevated to include physical violence between parents, the emotions and reactions of students became much more complicated, especially in the context of a step-family. For example, one girl described the experiences as ‘a real shock’ for her:

When my parents argued and fought with each other, I felt really scared. After that I felt unsafe and insecure when alone. I was afraid of someone coming to beat me. It affected my studying. … My family has a new member, my dad's child. It was unbelievable, sometimes I hate that child and am angry with my dad. … My family was already unhappy and it was a real shock for me. It had a great impact on my mental health and now I feel really sad and scared each time I have to go home and face my dad and the child. I feel like my dad loves the child more than his official [legal] family. Feelings of disappointment are always in my head. [17, female, rural]

Of concern is the students’ revelation of despair and suicidal behaviours upon being maltreated by their parents:

I feel that life is meaningless. My dad is very conservative. If I make even a minor mistake, he will yell at me and … beat me. I attempted suicide many times, but was not successful. [16, female, urban]

Violence from teachers was also reported as being discomforting and intimidating, with one young urban woman writing that ‘It's so uncomfortable when male or female teachers scold or beat their students’.

Students also described violence perpetrated by peers, who were referred to as ‘classmates’ and even ‘friends’. The emotional consequences of peer victimisation appear to be more serious than those of teacher victimisation. For some students, peer victimisation resulted in feelings of confusion about the explanations of such behaviour:

Why do classmates bully each other? Why have they cheated on other people for their own benefit? [18, male, rural]

Students described these experiences as betrayal and expressed hatred of peers once thought of as good friends. They wrote about feeling sad, stressed, and losing interest in studying, and about symptoms of depression, including poor sleep, loss of appetite, and even suicidal ideation.

There was a period of time I was hated and isolated by my friends. I hate those who were jealous. They told bad things and fabricated things about me which made me ashamed while I was considering them good friends and helped them with everything. But they turned their backs on me and isolated me. At that time I just wanted to die. Each day going to school I felt stressed, depressed, sad and did not want to study at all. I slept and ate very little because I was being obsessed with other people's eyes on me. [18, female, rural]

Some students experienced disturbing sexual assaults by strangers. One 16-year-old urban girl wrote that ‘two strange men riding past me grabbed and hurt my breast’. She added that such experiences made her feel ‘ashamed and self-pitying.’

When students reported multiple forms of victimisation (also termed poly-victimisation), they found the experiences to be much more detrimental. All described lack of interest in activities, diminished self-esteem, hatred of perpetrators, feelings of being insulted, isolated, and stressed, with suicidal thoughts as a means of escaping the situation or avenging the perpetrators. For example, one boy wrote:

My father often beats and scolds loudly even though I've grown up. With common mistakes or those which do not deserve a scolding, my father exaggerates them and beats me severely. He is often unreasonable, and a lot of the times it felt unbearable. … Sometimes he made me take a whole day off school just because of minor mistakes. … At school, sometimes my head mistress asked for my parents just because of minor, not serious mistakes. … The head mistress should be understanding and should not be too strict.

One girl wrote:

I feel that life is meaningless and worthless. … I have dark skin like my mother, am not as good-looking as other girls, and I am often teased, told bad things and denigrated by my friends. It destroys my self-esteem and makes me sad. Sometimes I am told bad things at home, especially by my mother. … For example, she said ‘You are kind of brain-wasted. You'd better die! [Go to hell]’. At times like that I often wanted to cry out loud or die, so that my mother could get rid of me. [16, female, rural]

The students not only had suicidal thoughts, they had attempted suicide. One girl had attempted suicide ‘many time’; she explained that:

My mum passed away and I want to follow her. Because she was the person I love the most and also the one who loved me the most. When she was still alive, she was also often beaten by my dad. [16, female, urban]

Poly-victims wrote about feeling isolated and socially disconnected, as did this young woman:

Even though I live in a family with all members (parents, two sisters, grandparents and uncle …), I find it hard to be open and share private or intimate things with my family members. Sometimes I feel lonely because my parents are not understanding. When I'm at school, I also feel sad because of arguments with and among friends. [16, female, urban]

Students gave evidence that experiencing violence had consequences other than adverse emotions, including diminished academic performance:

My parents have frequent arguments recently. … I was not hard-working, my academic results got much worse. [15, female, rural]

Violence also led to poor relationships with parents:

My dad is often angry although he does not beat me, but his irritation makes me uncomfortable. … My mum does not care what I want and need. … My parents rarely speak and discuss with me, thus they will not be able to understand me. [18, female, rural]

When both parents were experienced as unkind and unsympathetic, it could disrupt the adolescent's health both directly and indirectly. Students reported feeling emotionally disengaged and distant from their parents and afraid of sharing their problems. One young woman, for example, said:

My parents sometimes appear not to understand me. My mum has attitudes, behaviours and sayings that make me feel insulted. My dad was once so angry that he threw a remote control at me. I am suffering from an illness, but do not have the courage to tell my parents because I'm afraid my parents will scold at me and they will be sad. I feel bored with everything. There have been many times I was so mad at my family-related problems that I had thoughts of suicide. [15, female, urban]

### Theme 3: Hopes and aspirations in the context of victimisation

Students often expressed a yearning for an end to violence and the transformation of their family life so that all members could be happy:

My dad often drinks alcohol and each time when he is drunk, he comes home scolding, breaking and destroying things. Sometimes I feel really sad because happiness seems so vague to me. Only my mum works and earns money to raise me; thus [we] face economic difficulties. I wish my family would not face difficulties with family happiness and emotions anymore. [undisclosed age, female, rural]

Some students wanted more autonomy as well as better understanding from their parents. One girl wrote that she wanted more freedom, to be less restricted, and to have an ‘appropriate environment to study better’. In particular, she wanted her parents to be ‘understanding’ of her personal characteristics and desires. This need for understanding and appreciation was also implied in text written by other students about their family relationships, as in this example:

I have an unhappy family life. My mum left me to work overseas, my dad sometimes drinks alcohol and comes home yelling at me unreasonably; he recalls things in the very distant past and scolds me. He prohibits me in all sort of things. Once I arrived home 15 minutes late and he yelled and beat me right in the street. He stops me hanging out with my friends. … Sometimes I wonder why I have parents like mine? …I wish my dad did not drink alcohol and scold me, and would be a bit more easy-going with me. [16, female, urban]

Similarly, students wanted their teachers to be more understanding:

Sometimes teachers do not understand us at all, either. Every time when I was in the mood of studying, but suddenly being scolded at by my mother or the teacher, I felt a really low mood. … I want adults to be more understanding of us. [16, female, urban]

Students would welcome an adult, such as a teacher, to support them in peer conflicts. One 16-year-old urban girl wrote about ‘friends in my group who want to destroy my life and my image in other people's eyes’ and that she wanted ‘somebody to help me solve or deal with the conflicts I have at school.’

The need for help was especially strongly expressed by students who had experienced poly-victimisation:

Friends often tell bad things about each other. … Teachers often impose [their will on us], repress students, always try to appear fair, but are not in reality. … In my family, my mum always yells and scolds me severely. Although I am already in grade 11, I am still being slapped and scolded even though I did not do anything wrong. … I do not know what to do. [17, female, urban]

## Discussion

These data make an original contribution to knowledge about adolescents’ experiences of victimisation and its consequences for their lives. Although a limited proportion of participants provided free text, they included female and male students from different educational institutions in rural and urban areas in Vietnam.

### Experiencing multiple forms of violence

As reported elsewhere (Le *et al.* 2015), the survey component of this study found that over one-third of the participants had experienced more than ten of the 37 forms of victimisation assessed by the JVQ-R2. The elaborated accounts illustrated that victimisation did not occur in a single specific environment, but was pervasive, occurring at home, at school and in their communities. While more girls than boys provided elaborated accounts, the descriptions of these co-occurring experiences of violence were similar.

### Forms of victimisation not included in quantitative measures

The accounts revealed forms of victimisation by parents and teachers that were not included in the JVQ-R2, the standardised measure of violent victimisation. Abusive adults were often experienced as being extremely controlling, imposing prohibitions on friendships or autonomy, and as belittling and unfair. These findings may reflect the 1000 years Vietnam has experienced under the rule of different Chinese empires and Confucian ideology, in which children and adolescents are expected to respect and obey their parents and teachers.

The findings also illustrate differences among countries in terms of the forms of victimisation adolescents experience and their prevalence. This is consistent with previous research, which has also shown that certain forms of violence, including child maltreatment, are much more prevalent in some countries than in others (Akmatov, 2011). It has been documented in focus group discussions that standardised instruments can fail to capture the nature of some kinds of aggression or hostility (Marsh *et al.* 2007). Surveys limited to assessing universal forms of victimisation may thus fail to capture these country-specific forms.

### Serious consequences of violent victimisation

These data provide powerful accounts of the detrimental impacts of violent victimisation on the mental health and quality of life of adolescents. Participants described the corrosive effects of criticism and belittling on self-confidence; the loneliness of experienced parents and teachers as insensitive, coercive or threatening; the sadness of being socially excluded or ostracised; and how these accrued to reduce the sense that life was worthwhile and, at worst, to the contemplation of self-harm and death. Severity of psychological distress appeared to be worse if the perpetrator was a person on whom the adolescent relied (a parent or teacher) than a stranger, and among those who were directly victimised rather than witnesses. These findings echo those documented among young people in rural Canada who expressed feelings of being damaged and demeaned and had suicidal thoughts when being bullied, with psychological violence being considered ‘the most damaging to the recipient’ and increasing in severity in line with the closeness of the adolescent to the perpetrator (Kulig *et al.* 2006).

Exposure to violence also resulted in the adolescents’ perception of reduced academic performance. This has been recorded in a longitudinal survey of adolescents in the United States (Lepore & Kliewer, 2013). Poor academic performance may be attributable to the adolescents’ distraction from studying, loss of motivation, fear of the abusive parent, and emotional consequences of being maltreated. Sleep disturbance has also been suggested as an explanation for the association between exposure to violence and poor academic outcomes (Lepore & Kliewer, 2013).

These results also confirm the association between exposure to violence and mental health problems and suicidal ideation among adolescents found in the quantitative part of this research (Le *et al.* 2016*b*) and elsewhere (Nguyen *et al.* 2010; Le *et al.* 2017; Tran *et al.* 2017). They show the wide range of consequences of victimisation for adolescents; some of these consequences may not be captured by standardised instruments.

### Harsh child discipline, peer bullying and poor parent–child and teacher–student communication

This study also reveals the prevalence of harsh parenting practice, neglect and lack of parent–child understanding and of parent–child communication, which result in the adolescents’ perceptions of being maltreated. These findings are consistent with results reported by the United Nations Children's Fund (2014) that nearly 30% of adults worldwide believe that ‘physical punishment is necessary to properly raise or educate children’. These findings suggest that awareness of the adverse effects of harsh parental discipline and education about other parenting strategies may assist in preventing experiences of violence and victimisation among adolescents in Vietnam.

Bullied adolescents in this study expressed feelings of sadness, loss of self-confidence, and hatred of the peers who bullied them. This is consistent with results from previous research that peer bullying is associated with increased risk of suicidal thoughts among Vietnamese adolescents (Phuong *et al.* 2013). These experiences are universal, found among adolescents in both high- and low-income settings (Arseneault *et al.* 2010; Holt *et al.* 2015).

Lack of communication between adolescents and their teachers and guardians results in poor recognition or ignorance of peer victimisation by parents and teachers. A grade-7 female Vietnamese student was reported in the media as being beaten by her friends and seriously injured, yet it was not until 2 months later that her teachers and parents become aware of the incident (An Nhơn, 2015).

Many Vietnamese parents are afraid that adolescents’ relationships with a boyfriend or girlfriend may cause distractions, unsatisfactory academic results, and undesirable sexual behaviour. However, instead of open discussions, parents often prohibit adolescents from having such relationships. Although most parents in Vietnam rely on teachers to educate their children about sexual health, contraceptive use, and safe sex, sex education is often limited or non-existent. Teachers are often shy or hesitant about having such discussions with their students. Economic development, a busy life and higher levels of stress also result in parents spending less time with their children in Vietnam (Nguyen, 2006). This may result in poor parent–adolescent communication and relationships. Phuong and colleagues (Phuong *et al.* 2013) found that Vietnamese fathers’ ‘overprotection’ is associated with increased risk of suicidal thoughts among their daughters.

### Adolescents’ hopes and aspirations in the context of victimisation

Adolescents wrote explicitly about their need for help in dealing with victimisation and its consequences. Their comments suggested that those who might help them appeared to lack the knowledge and skills required to do so effectively. Despite growing awareness in Vietnam about violence, there are no official national programs to educate adolescents, parents, or teachers about violence. Misperceptions about what constitutes child maltreatment is evident among adolescents and teachers in Vietnam (Nguyen, 2006), with some types of physical maltreatment considered acceptable, reasonable, and even necessary. There are a few youth services in Vietnam, including the free Helpline for consultation and protection of children and adolescents, but adolescents, their teachers and guardians may not be aware of them. Although counsellors are available at some schools, there is little evidence of their effectiveness and of whether the counsellors have the appropriate skills to support victimised adolescents.

## Implications for policy and practice in Vietnam

These findings have important implications for policy and practice in Vietnam, including the need to raise community awareness about violence and appropriate discipline. Education and intervention programs to equip parents and teachers with the knowledge and skills to work with adolescents, to recognise the importance of violence prevention, and to improve parent–adolescent and teacher–student relationships should be established. Adolescents should also be educated to recognise violent acts, to avoid perpetrating peer victimisation, to deal with victimisation, and to protect themselves from being victimised. These are important in the prevention of violence among adolescents in Vietnam and to ensure their optimal development.

## Directions for future research

Studies using both quantitative and qualitative methods are recommended for research about exposure to violence among adolescents in order to establish a comprehensive understanding of adolescents’ experiences. Quantitative measures of violence against children and young people should include excessive parental control and teachers’ belittling or unfair treatment of students. The impacts of victimisation on other aspects of adolescents’ quality of life and wellbeing, such as social participation, should also be investigated. More qualitative research, including focus group discussions and in-depth interviews, with young people in Vietnam is warranted. Qualitative methods provide an opportunity for better understanding of adolescents’ experiences of and reflection on violence, which can inform more targeted prevention programs and respond more appropriately to young people's needs.

## Conclusions

Adolescents in Vietnam experience various forms of victimisation occurring at home, at school, and in the community, some of which may not be identified by standardised instruments. Exposure to violence is detrimental to adolescents’ health and wellbeing, often resulting in adverse emotions, and suicidal behaviours. It also affects adolescents’ academic performance and parent–child, teacher–student, and student–student relationships. Understanding adolescents’ experiences of and perspectives on violence and the impact it has on their health and wellbeing is important in the prevention of violence against adolescents in Vietnam.
